# Flexible Neural Probe Fabrication Enhanced with a Low-Temperature Cured Polyimide and Platinum Electrodeposition

**DOI:** 10.3390/s22249674

**Published:** 2022-12-10

**Authors:** João R. Freitas, Sara Pimenta, Diogo J. Santos, Bruno Esteves, Nuno M. Gomes, José H. Correia

**Affiliations:** 1 CMEMS—UMinho, University of Minho, 4800-058 Guimarães, Portugal; 2 LABBELS—Associate Laboratory, Braga/Guimarães, Portugal

**Keywords:** photolithography, thin-film deposition, passivation, electrodeposition, electrochemical impedance spectroscopy

## Abstract

Polyimide is an emerging and very interesting material for substrate and passivation of neural probes. However, the standard curing temperature of polyimide (350 °C) is critical for the microelectrodes and contact pads of the neural probe, due to the thermal oxidation of the metals during the passivation process of the neural probe. Here, the fabrication process of a flexible neural probe, enhanced with a photosensitive and low-temperature cured polyimide, is presented. Annealing tests were performed with metallic films deposited on polyimide, which led to the reduction of the curing temperature to 250 °C, with no significant irregularities in the metallic sample annealed at that temperature and an effective polyimide curing. The use of a lower curing temperature reduces the thermal oxidation of the metals during the polyimide curing process to passivate the neural probe. Additionally, in this fabrication process, the microelectrodes of the neural probe were coated with electrodeposited platinum (Pt), only after the passivation process, and its electrochemical performance was accessed. At 1 kHz, the impedance of the microelectrodes before Pt electrodeposition was approximately 1.2 MΩ, and after Pt electrodeposition, it was approximately 350 kΩ. Pt electrodeposition changed the equivalent circuit of the microelectrodes and reduced their impedance, which will be crucial for future in-vivo tests to acquire the electrical activity of the neurons with the fabricated neural probe.

## 1. Introduction

Neural interfaces are devices capable of recording or stimulating neural activity. Specifically, implantable neural probes with microelectrodes can record field potentials and single neuron activity with high temporal resolution (0.2–7 kHz) [1].

Silicon has been extensively used as the substrate for neural probe implementation, especially due to its rigidity and reproducibility during microfabrication. However, there is a high mechanical mismatch between silicon probes (~150 GPa Young’s modulus) and brain tissue (~1 kPa Young’s modulus), which increases tissue damage and immunological response and leads to gradual neural probe degradation. In the last years, flexible and polymeric-based neural probes have been developed to mitigate probe-brain mismatch and all its effects in an attempt to maintain the probe viability and to improve the quality of the recorded signals [1,2,3,4,5]. Some of the most common polymers used for neural probe implementation are polyimide, parylene–C, polycarbonate, epoxy, PDMS and SU-8. Young’s modulus is in the range of 1 MPa to 5 GPa [6]. Neural probe flexibility also presents a challenge during probe implantation in the brain, due to the strong possibility of probe buckling. Nonetheless, there are some strategies to temporarily increase the rigidity of polymeric probes, e.g., coating the probes with absorbable molecules that confer temporary rigidity or using mechanical carriers to guide them to the brain region of interest [6].

Polyimide neural probes can be implemented with photosensitive or non-photosensitive polyimide [6]. The implementation of a neural probe and its passivation with a non-photosensitive polyimide requires the use of O_2_/CF_4_ etching processes to define probe geometry and to expose the microelectrodes and contact pads of the neural probe [7,8]. On the other hand, the use of a photosensitive polyimide makes it possible to pattern the probe geometry and perform its passivation only with photolithographic and curing processes [3]. Nonetheless, the current photosensitive polyimides used to implement neural probes have a standard curing temperature of 350 °C, which leads to the thermal oxidation of the metals during the final curing process of the polyimide to passivate the neural probe [6,9,10].

Thus, this work presents the fabrication process of a flexible neural probe, achieved with an enhanced photosensitive and low-temperature cured polyimide. The use of a low-temperature cured polyimide reduces the thermal oxidation of the metals during the final curing process of the polyimide to passivate the neural probe. This work also presents the electrochemical characterization of the neural probe microelectrodes, which were coated with electrodeposited platinum (Pt), only after the neural probe passivation, reducing the possibility of thermal oxidation of the microelectrodes.

## 2. Materials and Methods

### 2.1. Microfabrication Process

The neural probe fabrication process consisted of four steps: (i) photolithography and curing processes to pattern the neural probe geometry; (ii) photolithography and titanium (Ti) thin-film deposition to pattern the contact pads, tracks and microelectrodes; (iii) photolithography and curing processes to passivate the neural probe; and (iv) Pt electrodeposition for the coating of microelectrodes.

**Figure 1:**
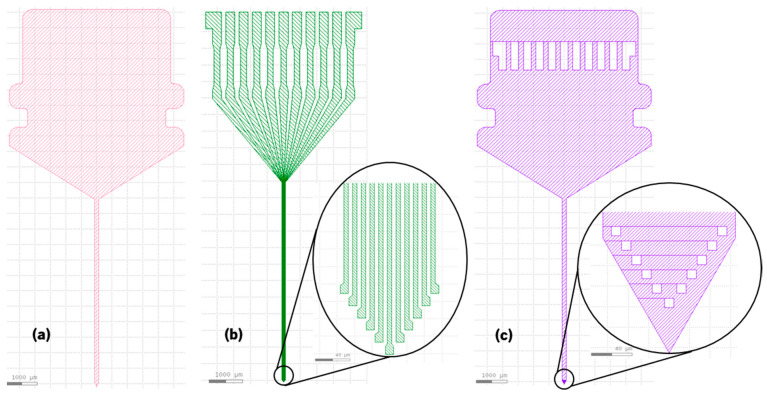
µMLA digital masks to pattern: (**a**) neural probe geometry; (**b**) neural probe contact pads, tracks and microelectrodes; (**c**) neural probe passivation.

Firstly, the photosensitive polyimide (LTC 9305 from Fujifilm) was patterned to define the neural probe geometry. Briefly, the polyimide was spin-coated on a silicon (Si) sacrificial slice. Then the slice was exposed to ultraviolet (UV) light using µMLA equipment (maskless aligner from Heidelberg), in which it is possible to digitally define a mask (Figure 1a). After UV exposure, the slice was immersed in a developer and rinse solutions (HTR-D2 and RER 600 from Fujifilm, respectively) to dissolve the unexposed polyimide. Finally, the polyimide was completely cured in a lab oven at 330 °C for 4 h, resulting in a 4-µm-thick layer of polyimide.

Secondly, the contact pads, tracks and microelectrodes were patterned on the neural probe. A 7-µm-thick negative photoresist layer (AZ nLOF 2070 from MicroChemicals) was spin-coated on the patterned polyimide. Then the sample was exposed to UV light, using the same µMLA equipment and a digitally defined mask (Figure 1b). After UV exposure, the sample was immersed in a developer (AZ 726 MIF from MicroChemicals) to dissolve the unexposed photoresist. Afterward, a 100-nm-thick Ti thin-film was deposited by electron beam (7 kV/60 mA), with a deposition rate of 1.6 Å/s. Then the sample was immersed in a stripper (TechniStrip NI555 from MicroChemicals), removing the remaining photoresist from the lift-off process.

Thirdly, the neural probe was passivated with the same photosensitive polyimide. Once again, the polyimide was spin-coated on the sample. Then the sample was exposed to UV light using the same µMLA equipment and a digitally defined mask (Figure 1c). Following that, the sample was immersed in the same developer and rinse solutions to dissolve the unexposed polyimide. Finally, the polyimide was completely cured in a lab oven at 250 °C for 4 h, leaving only the contact pads and microelectrodes exposed. The lower curing temperature was used to reduce the probability of Ti oxidation. The used polyimide (LTC 9305 from Fujifilm) is an NMP (N-Methylpyrrolidone)/NEP (N-Ethylpentedrone) and halogen-free formulation with an appropriate curing temperature between 200 °C and 380 °C, contrarily to the currently used photosensitive polyimides to implement neural probes, which have a standard curing temperature of 350 °C [6,9,10].

Finally, Pt electrodeposition was performed to coat the microelectrodes of the neural probe. Pt electrodeposition was performed using a solution of 2 × 10^−3^ mol/dm^3^ K_2_PtCl_6_ and 0.1 mol/dm^3^ HClO_4_ and with a three-electrode configuration: neural probe microelectrode as the working electrode; a Pt foil as the counter electrode; and a Hg/Hg_2_SO_4_ electrode as the reference electrode [11]. A potentiostat was used (Reference 600, Gamry Instruments) to perform a chronopotentiometry with a current density of −1167 µA/cm^2^. It is also relevant to refer that to access the viability of the Hg/Hg_2_SO_4_ reference electrode; its impedance was acquired previous to Pt electrodeposition.

**Figure 2:**
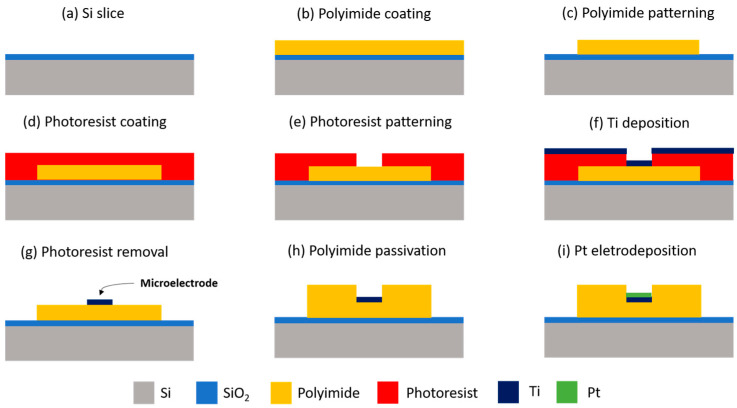
Overview of the neural probe fabrication process. The neural probe release from the sacrificial Si slice is achieved by wet etching of the sacrificial SiO_2_ thin-film, which is deposited between the Si and the polyimide.

A general overview of the complete neural probe microfabrication process is shown in Figure 2.

### 2.2. Electrochemical Impedance Spectroscopy Measurements

Electrochemical impedance spectroscopy (EIS) measurements were performed before and after Pt electrodeposition. EIS measurements are crucial to the fabricated neural probe characterization, due to its main purpose of being used in optogenetics studies, where an optical source is used for neuron stimulation and the microelectrodes are used to record the optically induced neural activity. A low impedance is the main indicator of a microelectrode ability to record neural activity [12].

EIS was performed using a saline solution (0.9% NaCl) and with a three-electrode configuration: a neural probe microelectrode as the working electrode; a Pt foil as the counter electrode; and an Ag/AgCl electrode as the reference electrode. A potentiostat (Reference 600, Gamry Instruments) was used, and an AC potential of 10 mV was applied, between the counter and the working electrodes, with frequencies ranging from 1 MHz to 100 Hz. It is also relevant to refer that, to access the viability of the Ag/AgCl reference electrode, its impedance was also acquired previous to EIS measurements in the neural probe.

## 3. Results and Discussion

**Figure 3:**
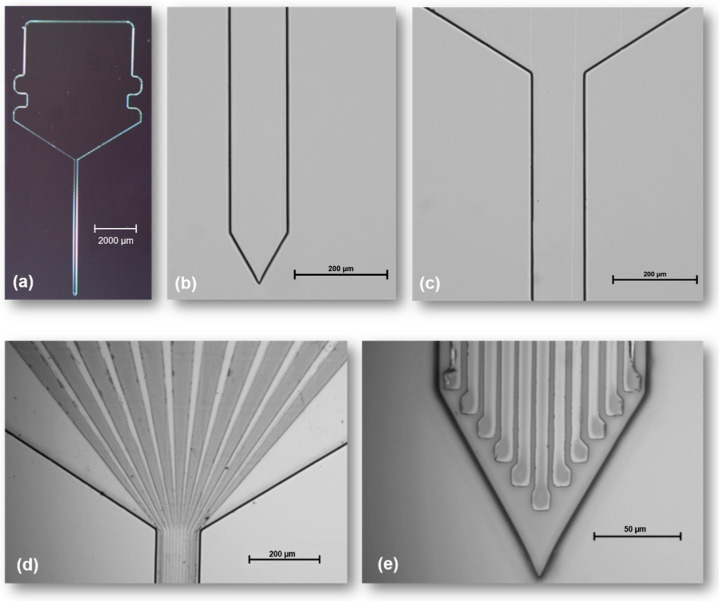

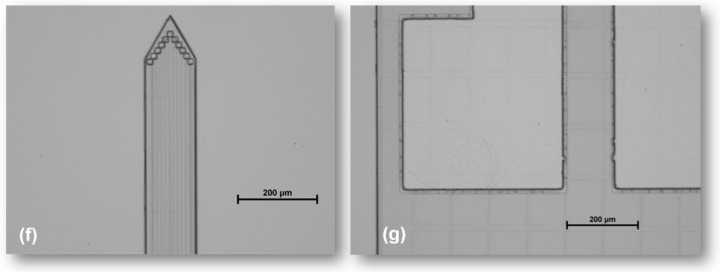
Fabricated neural probe: (**a**–**c**) patterning of the neural probe geometry; (**d**,**e**) patterning of the contact pads, tracks and microelectrodes; (**f**,**g**) passivation of the neural probe, leaving the microelectrodes and contact pads exposed.

Figure 3 shows microscope images of the fabricated neural probe. The final dimensions of the neural probe are approximately a 130-µm width, a 6-mm shaft length and a 10-µm thickness. The width and pitch of the tracks are 5 µm, and the microelectrodes have an active area of approximately 9 × 9 µm^2^.

**Figure 4:**
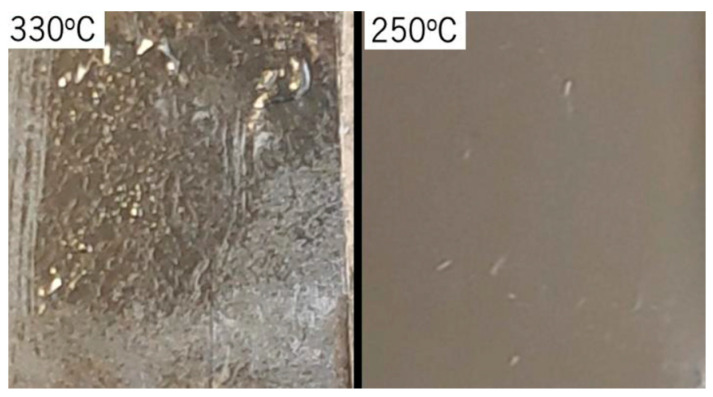
Metallic films were deposited on the polyimide after the annealing process.

During the fabrication process, several tests were performed to achieve the best results. One of the most relevant tests was to find the best polyimide curing temperature to define probe geometry and to perform neural probe passivation. For neural probe geometry definition, it was decided to keep a high curing temperature (330 °C) since this process is performed before patterning the microelectrodes, contact pads and tracks, and that way does not affect metal oxidation. For the neural probe passivation, since the metals are already patterned on the probe substrate, it is important to reduce the polyimide curing temperature. That way, a test was performed with two samples containing a metal stack (Ti, aluminum (Al) and Pt) deposited on polyimide. The two samples were subjected to two different annealing processes, one at 250 °C and the other at 330 °C, both for 4 h. Each sample after the annealing is shown in Figure 4. As can be seen, the temperature reduction to 250 °C had a positive effect in reducing the formation of bubbles and defects in the metallic films, with no significant irregularities in the sample annealed at 250 °C. After this test, it was decided to fix the polyimide curing temperature to 250 °C for the passivation process. To even reduce the microelectrode thermal oxidation probability, it was decided to electrodeposit Pt in the microelectrodes, only after the neural probe fabrication and complete passivation.

**Figure 5:**
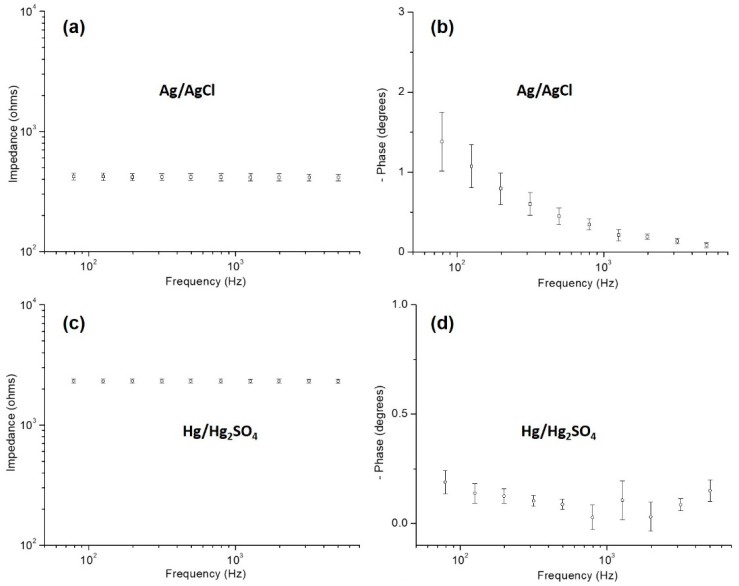

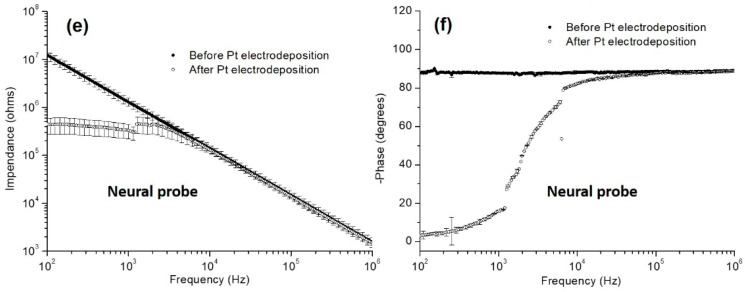
EIS measurement results for: Ag/AgCl reference electrode (**a**,**b**), Hg/Hg_2_SO_4_ reference electrode (**c**,**d**) and neural probe microelectrodes (**e**,**f**).

EIS measurements were performed before and after the Pt electrodeposition to evaluate the effects of electrodeposited Pt on the impedance values of the microelectrodes. The plots obtained are shown in Figure 5, in Bode format, with impedance plotted against frequency and with phase angle plotted against the frequency. Firstly, EIS measurements of the Ag/AgCl and Hg/Hg_2_SO_4_ reference electrodes were performed, as can be seen in Figure 5a–d, to make sure that their impedance and phase angle values were within the expected range for an optimum potentiostat performance. Then EIS measurements were performed with the fabricated neural probes, using the Ag/AgCl reference electrode. Pt electrodeposition was performed using the Hg/Hg_2_SO_4_ reference electrode. Right after the Pt electrodeposition was finished, new EIS measurements were performed with the fabricated neural probes, so that the before and after electrodeposition values could be compared—Figure 5e,f.

As can be seen in Figure 5a–d, EIS values for the Ag/AgCl and Hg/Hg_2_SO_4_ reference electrodes are in the range of acceptable values. The impedance values of reference electrodes must be lower than 5 kΩ (preferably lower than 1 kΩ) and the phase angle values should be near zero, especially for high frequencies [13].

In Figure 5e,f, the change of the impedance and phase angle values with the variation of frequency for the microelectrodes of the neural probe can be read. Analyzing the spectra, before the Pt electrodeposition, the microelectrodes exhibited a capacitive behavior; the impedance increases with the decrease of frequency; and the phase shift (Φ) is approximately 90°, which is in accordance with literature reports for Ti microelectrodes [14]. After Pt electrodeposition, the impedance curve starts to flatten at around 3 kHz, meaning that the capacitive behavior of the microelectrodes changes to resistive behavior. The phase angle is also in accordance with this change of behavior, starting at 0° and increasing to 90°. Thus, the equivalent circuit could be represented by a constant phase element (CPE) [15]. For neural recording applications, the most relevant frequency is around 1 kHz, due to the mean duration of a spike being 1 ms [16]. Before the Pt electrodeposition and at 1 kHz, the impedance of the neural probe was around 1.2 MΩ; after the Pt electrodeposition, the impedance of the neural probe decreased to around 350 kΩ. As expected, the Pt electrodeposition decreased the neural probe microelectrodes’ impedance significantly, which is beneficial for the recording of neural activity. The obtained impedance values of the Pt microelectrodes are also in the range of the literature values for Pt microelectrodes with reduced active areas [17], and also for other types of neural microelectrodes with reduced areas (e.g., gold (Au) microelectrodes) [7].

## 4. Conclusions

This work presented an improved fabrication process of a flexible and polyimide-based neural probe. A photosensitive and low-temperature cured polyimide was used to reduce the probability of metallic thin films’ thermal oxidation, during the polyimide curing process to passivate the neural probe. Annealing tests were performed with metallic films deposited on the polyimide, which led to the reduction of the polyimide curing temperature from 350 °C to 250 °C, with no significant irregularities in the metallic sample annealed at 250 °C.

Additionally, Pt electrodeposition was performed to coat the neural probe microelectrodes (Ti microelectrodes), only after the neural probe passivation process. The electrochemical performance of the neural probe microelectrodes was accessed to prove the viability of the fabrication process and future *in-vivo* tests with this flexible neural probe. Pt electrodeposition reduced the microelectrodes’ impedance from around 1.2 MΩ to 350 kΩ, which improves the capability of the neural probe to record the electrical activity of the neurons.

## Data Availability

Not applicable.
